# Retrospective Cohort Study of 4783 Morse Taper Hybrid Dental Implants: Survival Rate Analysis

**DOI:** 10.3390/bioengineering12121305

**Published:** 2025-11-28

**Authors:** Kleryo Câmara, Alexandre Negretto, Luiz Henrique Pegorini, Geninho Thomé, Sergio Rocha Bernardes, Tatiana Miranda Deliberador

**Affiliations:** 1 Department of Implantology, Instituto Latino Americano de Pesquisa e Ensino Odontologico College, Rua Jacarezinho 656, Curitiba 80810-130, PR, Brazil; kleryocamara@gmail.com (K.C.); dracn@terra.com.br (A.N.); geninho.thome@neodent.com (G.T.); sergio.bernardes@neodent.com (S.R.B.); 2 Independent Researcher, Curitiba 80810-130, PR, Brazil; hpd2011@hotmail.com

**Keywords:** dental implants, complications, survival rate

## Abstract

This retrospective study aimed to evaluate the survival rate of hybrid dental implants in different patient profiles and clinical conditions. A total of 1215 patients’ files were analyzed from patients with at least one hybrid dental implant inserted at ILAPEO College (Curitiba, Brazil) from 2018 to 2024. The data collection was performed from 2021 to 2024. Parameters related to patients, implants, and surgical characteristics were collected. Descriptive summary statistics were estimated for all parameters. The associations between the dependent variables “implant survival” and patient, procedure, and implant characteristics were assessed by the Cox proportional hazards model. A total of 4783 hybrid dental implants (Helix, GM, Neodent) were placed in 1215 patients with a mean age of 57.17 ± 12.09 years. The most frequent patients’ medical conditions were diabetes, hypertension, thyroid dysfunction, use of steroids (corticoids), psychological limitations, and bruxism and clenching. Patients were followed for a mean period of 29.54 ± 18.95 months. Immediate loading was applied in 2302 (48.13%) implants and conventional loading in 1735 (36.27%). One hundred and fifty-one implants were lost due to a lack of osseointegration, resulting in an implant survival rate of 95.4% (CI: 94.4%; 96.6%). Adverse events were reported in 389 (8.13%) implants. Uncontrolled hypertension, hypertension without information on control, absence of final abutment, replacement implant, and adverse event occurrence were associated with implant loss. Treatment using a hybrid macrogeometry dental implant is an option for total or partial edentulous patients with compromised health and different clinical conditions.

## 1. Introduction

Since Branemark discovered titanium osseointegration in the 1969s, dental implants have become the primary treatment for totally or partially edentulous patients with various clinical conditions. With decades of use, dental implants present high survival rates from 91.69% to 100% in up to 20 years of follow-up [1,2,3,4].

Despite scientific evidence on high implant survival rates and all the efforts from manufactures to prevent them, eventual failures occur. Commonly, implant loss is divided chronologically into early and late failure [5]. Early failures group all implants removed before prosthetic rehabilitation and late failures occur after occlusal loading [6]. Over time, multiple risk factors have been implicated in implant failure. For systematic analysis and interpretation, these factors are conventionally categorized into three primary domains: implant-related, patient-related, and surgically related factors [7].

Implant-related factors encompass variations in macrogeometry and prosthetic interface designs, both of which are continuously refined through ongoing advancements in the industry [8]. The initial implant macrogeometry was cylindrical, introduced by Branemark, and demonstrated long-term survival and success [9]. As macrogeometry evolved, tapered implants were introduced to enhance primary stability, particularly in poor-quality bone. The survival of both implants is comparable [10,11].

To potentiate the advantages of cylindrical and tapered implants, a hybrid macrogeometry was developed with a coronal cylindrical and taper apical part [12]. In parallel with advancements in macrogeometry, the design of the implant–abutment interface has also undergone significant evolution. The implant–abutment connection is the most critical part of the implant system because it must resist maximum masticatory forces and bacterial infiltration [13]. A greater mismatch between the diameters of implant and abutment, as seen in morse taper implants, leads to better bone preservation [14]. Even though no differences in implant success rate between platform matching and platform switching were reported, implants restored by platform switching showed a lower degree of marginal bone loss over time [15].

Patient-related factors continue to be critical determinants of implant survival. Lower implant survival rates have been correlated with several patient-related factors, including male sex [5,7], smoking [5,16], a history of treated generalized periodontitis [16,17], history of periodontal disease without supportive therapy in cases involving sinus floor augmentation [18], previous implant loss [16], uncontrolled diabetes mellitus [19], and intravenous antiresorptive therapy [19,20].

Surgical factors also play a pivotal role in implant survival. Variables such as complications registered during implant surgery [17]; implant placement in anterior regions [7] and in completely edentulous arches [5]; and implants placed by dental surgeons with less than five years of experience [21] may increase the risk of implant failure.

As implant survival remains influenced by implant design, as well as patient- and surgical-related factors, it is crucial to evaluate mid-term survival rates and determine the impact of these variables on implant loss. This study aimed to evaluate the mid- to long-term survival of morse taper hybrid dental implants, examining the influence of implant design, patient factors, and surgical variables on implant loss in a cohort of 1215 patients and 4783 implants.

## 2. Materials and Methods

### 2.1. Study Design and Data Collection

This study was approved by ILAPEO College ethical committee (process number: 6.792.960). Informed consent was waived, as this was a retrospective study based on clinical records. All data were collected and stored anonymously, without any individual identification, ensuring patient confidentiality and privacy.

The manuscript was prepared according to the Strengthening Reporting of Observational Studies (STROBE) in Epidemiology [22]. The data were retrospectively collected from clinical records of patients with at least one Helix GM implant (Neodent, Curitiba, Brazil) inserted at ILAPEO College (Curitiba, Brazil) from 2018 to 2024. All patients rehabilitated with Helix GM implants at ILAPEO until the date of this study were included in this sample. No exclusion criteria were applied. The data collection was performed from 2021 to 2024. No sample size was calculated since all medical records available were reviewed.

Three previously trained reviewers performed data extraction in parallel, dividing the clinical records among themselves. They were trained in standardization on identifying relevant information, understanding terms, and properly completing the electronic spreadsheet, which included flags to minimize data entry errors. Periodic audits of sample records were conducted to verify data consistency and correct any discrepancies. The following parameters were retrieved from patients’ files:

- Patient-related: age, gender, presence of comorbidities, smoking habits, oral hygiene, previous head/neck radiotherapy, and bruxism and clenching presence.
- Implant- and surgical procedure-related: implant length and diameter, prosthetic interface, bone graft procedure, tissue graft procedure, bone type, implant loading type, insertion torque, flap or flapless surgery, guided surgery, region of implant placement, adverse events, and implant survival.

As this is a retrospective study, data were obtained from clinical records filled out by different operators, without prior standardization of records. To ensure consistency between data, standardization was carried out during collection, using uniform criteria and terms for all analyzed variables. In this way, information was extracted from the medical records and reclassified consistently, ensuring comparability between records and feasibility for statistical analyses.

Information on systemic arterial hypertension and diabetes mellitus was obtained retrospectively from clinical records, based on medical anamnesis and health history records. The presence of these conditions was recorded according to the patient’s self-report and/or previous medical diagnosis noted in the medical record. The classification of each condition as controlled or uncontrolled was carried out according to the operator’s description, considering the regular use of medication and the clinical control reported at the time of care. When the medical record did not contain sufficient information for this classification, the variable was recorded as “not informed”.

Smoking status was extracted from the clinical records and categorized as smoker (>10 cigarettes/day), smoker (<10 cigarettes/day), former smoker, or non-smoker. Information on total smoking exposure (pack-years) was not consistently available.

Guided bone regeneration (GBR) was recorded as “performed” or “not performed” based on information from the clinical records. In the same way, when the medical record did not contain sufficient information for this classification, the variable was recorded as “not informed”.

Insertion torque was recorded and categorized in clinical records (<35 N·cm and ≥35 N·cm). The cutoff point adopted follows literature recommendations and implant instruction for use as a clinical threshold for adequate primary stability. The torque measuring devices used in the clinic follow usage routines according to the manufacturer’s instructions, respecting the recommended service life and frequency of use.

Surgical procedures were performed by multiple experienced surgeons and graduate students under the direct supervision of specialist teachers, following standardized clinical protocols. After implant placement, post-operative instructions, appropriate medication prescriptions, and scheduled follow-up appointments were given. The loading protocol was determined based on the date of implant placement and the date of prosthesis placement, as recorded in the clinical records. Cases were classified into two categories: immediate loading (up to 7 days after implant placement) and conventional loading. The type of prosthetic rehabilitation was categorized as single-unit, multi-unit, or full-arch.

The implant loss was determined from information recorded in clinical records. An implant was considered lost when the record indicated implant removal or mentioned osseointegration failure, clinical mobility, persistent pain, or uncontrolled peri-implant infection. The medical records were completed by different operators, following the university’s institutional standard, in which implant loss is usually defined as the need for removal due to lack of osseointegration, detectable clinical mobility, persistent pain, uncontrolled peri-implant infection, or radiographic bone loss that compromises stability.

In this study, no radiographic evaluation or measurement of marginal bone loss was performed, as data were collected retrospectively and were based exclusively on available clinical information.

### 2.2. Data Analysis

All analyses were performed using R software version 4.5,1, ensuring full reproducibility through automated scripts. Different packaging was used for each analysis: handling and cleaning—dplyr, tidyverse, lubridate; survival analysis—survival, survminer, coxme; visualization—ggplot2; missing treatment—naniar, mice; and export and results—broom, readr.

Descriptive summary statistics were estimated for all parameters. Quantitative parameters were described by mean, standard deviation, minimum, and maximum. For qualitative variables, frequencies were given.

Survival rate was estimated by the Kaplan–Meier estimator. For this analysis, time zero was defined as the date of implant placement. Censorship was applied to the last recorded follow-up for implants without loss. The event was defined as implant loss. Early failures were classified as those occurring within 6 months, and late failures as those occurring beyond 6 months after placement.

The association between the dependent variable “implant survival” and patient, procedure, and implant characteristics were evaluated by the Cox proportional hazards model. A Cox model that considered the dependence between implants from the same patient was applied with the patient cluster adjustment. It was not possible to adjust a frailty model per operator, as there is no corresponding variable in the database.

To address missing data, a table and a visualization map (Table S1 and Figure S1) were created to analyze the data with high missing rates. These variables were excluded from multivariate analyses due to low completeness and risk of bias.

To reduce exclusion bias (listwise deletion), we implemented multiple imputations by chained equations (MICE) with predictive mean matching and 20 imputed sets. Cox models were re-estimated in each set and combined (pooled estimates). Hazard Ratios and confidence intervals remained consistent with the full case results, confirming the robustness of our inferences about the main predictors. Finally, a correction for multiple testing using the Benjamini–Hochberg procedure (FDR) was applied for all exploratory bivariate comparisons between predictors and implant loss. The significance level for all tests was *p* < 0.05.

## 3. Results

### 3.1. Population Characteristics

The sample consisted of 1215 patients, of whom 740 (60.91%) were women and 475 (39.09%) were men, with a mean age of 57.17 ± 12.09 years (ranging from 24 to 93 years). The most frequent patients’ medical condition was controlled or uncontrolled diabetes (99 patients), controlled or uncontrolled hypertension (346 patients), controlled or uncontrolled thyroid dysfunction (101 patients), use of steroids (corticoids) (73 patients), psychological limitations (78 patients), and self-reported bruxism and clenching (71 patients). Presence of a weak immunological system (5; 0.41%), coagulation disorders (24 patients), unsuitable soft tissue capacity (16 patients), periodontitis (13 patients), previous head/neck radiotherapy (3 patients), and poor oral hygiene (7 patients) were presented in lower quantity either due to non-existence of these comorbidities or lack of information. Complete descriptive data are present in Table S2.

### 3.2. Procedure and Implant Characteristics

Regarding the surgical procedure, 1295 (27.08%) implants received bone grafts. Most bone graft procedures (927; 19.38%) occurred in conjunction with implant placement. The tissue graft procedure was performed 809 (16.91%) times, and the majority (420; 8.99%) in conjunction with implant placement.

Forty-six (0.96%) implants were placed in bone type I, 215 (4.50%) in bone type II, 232 (4.85%) in bone type III, and 44 (0.92%) in bone type IV; however, missing data was present in 4246 (88.77%) implants. Immediate loading was applied in 2302 (48.13%) implants and conventional loading in 1735 (36.27%). Most implants were placed with an insertion torque between 32–60 N·cm (3212; 67.18%). Nine (0.19%) implants were placed through flapless surgery, and 44 (0.92%) through an open flap. Guided surgery was used in 474 (9.91%) implants.

The main region of implant placement on the maxilla was premolar (823; 17.21%), followed by molar (702; 14.68%), incisor (530; 11.08%), full arch (218; 4.56%), and canine (174; 3.64%); 9 (0.19%) implants were not specified. On the other hand, the main region of implant placement on the mandible was molar (1120; 23.42%), followed by full arch (607; 12.69%), premolar (461; 9.64%), incisor (113; 2.36%), canine (29; 0.61%), not informed (3; 0.06%), and symphysis (1; 0.02%). Complete descriptive data are presented in Table S3.

**Figure 1:**
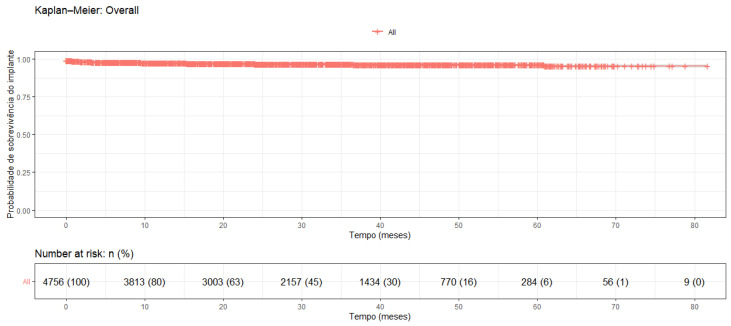
Kaplan–Meier survival analysis.

A total of 4783 Helix GM implants (Neodent, Curitiba, Brazil) were placed. Their length ranged from 8 to 18 mm, and their diameters from 3.5 mm to 7 mm. Almost all implants had a hydrophilic surface (Aqcua, Neodent, Curitiba, Brazil) except for one (0.02%) with a hydrophobic surface (Neoporos, Neodent, Curitiba, Brazil). Patients were followed for a mean period of 29.54 ± 18.95 months (ranging from 0 to 81.70 months). One hundred and fifty-one implants were lost due to a lack of osseointegration, resulting in an implant survival rate of 95.4% (CI: 94,4%; 96.6%) (Figure 1).

Adverse events were reported in 389 (8.13%) implants. The main adverse events reported were pain (55; 14.58%), marginal bone loss (55; 14.58%), paresthesia (42; 11.14%), and contamination (34; 9.01%).

### 3.3. Association Between Patient, Procedure, and Implant Characteristics with Implant Loss

A statistically significant association was found between implant loss and the presence of an adverse event (*p* ≤ 2 × 10^−16^), replacement implant (*p* = 0.002), uncontrolled hypertension (*p* = 0.039), hypertension without information on whether controlled (*p* = 0.017), and absence of final abutment (*p* = 0.002). Table 1 describes these associations, and Table 2 describes the models’ performance.

Patients with adverse events have a 19 times higher risk of failure (event) compared to those without. This is the strongest factor in the model. In the same way, replaced implants have a 2.2 times greater risk of failure than non-replaced implants. Regarding uncontrolled hypertension, it more than doubles the risk of failure compared with controlled hypertensives (or reference category). Hypertension without information on whether controlled appears to have an 86% lower risk than the reference group, but this may reflect missing data or misclassification, as the result is counterintuitive. Implants without abutments have a 24 times higher risk of failure.

The Schoenfeld proportional hazards test indicated a mild violation of the proportional hazards assumption (global test: *p* = 0.036), with evidence of non-proportionality for the adverse event (*p* = 0.020) and final abutment (*p* = 0.036) variables. These violations suggest that the effect of these variables varies over time, possibly being more intense in the initial follow-up periods.

## 4. Discussion

Implant-supported prostheses are a good choice for treating totally or partially edentulous patients. Indeed, this study found a high implant survival rate (96.83%) in a follow-up period of up to 6.8 years, showing that morse taper hybrid dental implants are a reliable option for patients with comorbidities and different clinical conditions.

Implant selection influences the process of osseointegration, initial mechanical stability, and the long-term clinical success of the treatment. Taper implants are indicated for low bone density or for recent extraction sockets [23]. Due to the combination of taper and cylindrical shapes, hybrid macrogeometry can be used for all bone types, facilitating clinician practice. Indeed, this study demonstrates that the hybrid implant used has high implant survival and is safe in all bone types and for different clinical conditions.

The survival rate of cylindrical and tapered implants has been extensively studied. Studies observed survival rates between 81% and 98.7% in up to 10 years of follow-up [9,24,25,26]. One study evaluated a hybrid implant with a similar design to the Helix GM implant and observed a survival rate of 92% to 98.6% in a 1-year follow-up when subjected to different loading and insertion protocols [12]. These survival rates are similar to our findings. However, our study highlights some important topics. The survival rate found in our study was evaluated in a high quantity of implants. In addition, the implants were placed in a diverse population with different clinical conditions and by multiple clinicians, including non-experienced, and even in this scenario, the survival rate was high.

Many studies describe changes in design as one factor improving implant stability [27]. Indeed, the hybrid implant used in this study was designed to achieve high insertion torques and allow immediate loading. More than 70% of the implants evaluated in this study achieved insertion torques higher than 32 N·cm, allowing immediate loading. These findings support the clinical application of the hybrid macrogeometry implant design in immediate loading protocols.

Health-compromised patients are a challenge. It is crucial to identify potential risk factors associated with implant failure and assess their manageability. The association analysis of patient characteristics and implant loss in this study showed an association between hypertension and implant failure. Some patients’ characteristics as a risk factor for implant failure have been extensively discussed, and there is a controversy in the literature. Hypertension was associated with implant loss. However, a few studies evaluating this influence and a systematic review found no association between hypertension and dental implant failure [28].

Multiple studies in the literature evaluated the influence of dental implants placed in sites of previously failed implants. Chrcanovic et al. [29] assessed 175 replaced implants in a population of 10,096 implants and found a statistically significant difference in the survival rate between implants that were inserted for the first time (94%) and implants that replaced the ones lost (73%). Another study demonstrated that the survival rate decreases with subsequent reimplantation [30]. These data corroborate the association between replaced implants and implant loss found in this study.

The presence of adverse events was associated with a 19 times higher likelihood of implant failure, and the effect of this variable varies over time, possibly being more intense in the initial follow-up periods. This is similar to what is found in the literature, where surgical complication was significantly associated with early implant failure, with a 15.84 times higher likelihood of implant failure [31]. Another study found that infection may be considered a risk factor for early failure of osseointegrated implants [32]. It can be inferred that the occurrence of adverse events, mainly in the initial phase of osseointegration, can be determinant in the implant survival.

Since this study is retrospective, missing data could result from poor registration quality or variables not considered registered in advance. In both cases, the origin of missing information can lead to information bias. Analyses of the correlation between patient characteristics and parameters of interest may also minimize confounding bias. Additionally, missing or uninformed data were treated to prevent compromising the results. Another limitation inherent to retrospective design is the lack of information due to the clinician not reporting adequately in the patient file, leading to a conclusion that differs from the actual scenario. In this way, variables with low information must be evaluated with caution.

Torque data were recorded only in categories, making it impossible to present the continuous distribution of values. Furthermore, it was not possible to retrospectively identify the specific models of the devices used for torque measurement, although the clinic follows standardized usage routines as recommended by the manufacturers.

Additionally, this study did not allow for the assessment of inter-operator variability, as the individual identification of each surgeon was not recorded in the medical records. Although the procedures followed standardized protocols and were directly supervised, the influence of operator experience on the results cannot be completely ruled out. Another significant limitation is the lack of radiographic evaluation of the implants, as failure determination was based solely on explicit records of loss in the medical records.

In this way, prospective studies are needed that include standardized radiographic measurements of MBL, allowing for more detailed comparisons. It is also advisable to consider continuous torque recording and detailed documentation of the devices used, allowing for more comprehensive analyses.

## 5. Conclusions

Within the limitations of this single-center retrospective study using a specific implant system (Helix, GM, Neodent), the hybrid macrogeometry evaluated demonstrated survival rates suggesting it may be an option for patients with different clinical conditions, including those with systemic health compromises. The implant survival rate was 96.83% in up to 6.8 years of follow-up. A low complication rate of 8.13% was observed, with the majority of adverse events classified as mild and manageable.

## Figures and Tables

**Table 1:** Association analysis of variables at implant level (n = 4783).

Variable	Model 1		Model 2	
	Hazard Ratio	*p*-Value	Hazard Ratio	*p*-Value
Adverse event = yes	19.22 (11.8–31.3)	<0.001	19.10 (12.0–30.5)	<0.001
Cover screw = yes	0.48 (0.24–0.94)	0.03	0.63 (0.35–1.11)	0.11
Bone graft procedure = yes	2.2 × 10^−7^ (1.2 × 10^−8^–4.1× 10^−6^)	<0.001	—	—
Region of placement on mandible	HRs 10^6^–10^7^	<0.001	—	—
Replacement implant = yes	2.06 (1.19–3.56)	0.010	2.24 (1.34–3.76)	0.00
Use of healing abutment = yes	0.57 (0.36–0.93)	0.023	—	—
Hypertension = no	0.52 (0.33–0.81)	0.004	0.83 (0.49–1.41)	0.50
Hypertension = uncontrolled	1.63 (0.69–3.86)	0.264	2.55 (1.05–6.21)	0.04
Hypertension = yes, not informed	0.11 (0.02–0.50)	0.005	0.14 (0.03–0.70)	0.02
Final abutment = GM Mini Conical	1.65 (0.20–13.7)	0.64	1.83 (0.23–14.6)	0.57
Final abutment = no abutment	18.6 (2.4–143.0)	0.005	24.0 (3.1–186.0)	0.00
Final abutment = standard abutment	4.26 (0.53–34.2)	0.17	5.10 (0.64–40.6)	0.12
Smoking	HRs 10^6^–10^7^	<0.001	—	—
Other disease = yes	0.90 (0.57–1.41)	0.64	—	—

**Table 2:** Description of each model’s performance.

Metric	Model 1	Model 2
N (observations)	4	4.46
Events	122	134.00
Concordance (C-index)	0.932	0.92
Likelihood Ratio Test	452.4 (24 df), *p* < 0.001	470.9 (9 df), *p* < 0.001
Wald Test	4989, *p* < 0.001	303, *p* < 0.001
Score (robust)	75.7, *p* = 3 × 10^−7^	70.1, *p* = 1 × 10^−11^

## Data Availability

The original contributions presented in the study are included in the article/Supplementary Material, further inquiries can be directed to the corresponding author.

## Supplementary Materials

The following supporting information can be downloaded at: https://www.mdpi.com/article/10.3390/bioengineering12121305/s1, Figure S1: Missing data map; Table S1: Analysis of missing data; Table S2: Descriptive analysis of the patient’s characteristics at the patient level (n = 1215); Table S3: Descriptive analysis of surgical procedure variables at implant level (n = 4783).
