# Supplementary material for: Retrospective Cohort Study of 4783 Morse Taper Hybrid Dental Implants: Survival Rate Analysis
Source: Bioengineering (Basel). 2025 Nov 28;12(12):1305. doi: 10.3390/bioengineering12121305 (PMC12729377; doi:10.3390/bioengineering12121305)
Supplement: Supplementary file 1 [file bioengineering-12-01305-s001.zip › bioengineering-3881202-supplementary.pdf]

Table S1. Analysis of missing data.

Variable	Missing	Total	Percentage
Cooperative and motivated patient	4778	4783	99,90%
Weak immunological system	4758	4783	99,48%
Presence of poor oral hygiene	4737	4783	99,04%
Periodontites	4730	4783	98,89%
Flapless or_ open flap surgery	4730	4783	98,89%
Implant loss date	4633	4783	96,86%
Thyroid disfunction	4372	4783	91,41%
Bruxism or clenching	4327	4783	90,47%
Bone type	4246	4783	88,77%
Unsuitable soft tissue healing capacity	2509	4783	52,46%
Coagulation disorders	2501	4783	52,29%
Pregnancy	1443	4783	30,17%
Straight/angled final abutment	1066	4783	22,29%
Insertion torque (n.cm)	672	4783	14,05%
Smoking	224	4783	4,68%
Final abutment	196	4783	4,10%
Regular use of steroids	193	4783	4,04%
Previously head/neck radiotherapy	183	4783	3,83%
Psychological limitations	124	4783	2,59%
Hypertension	104	4783	2,17%
Diabetes	91	4783	1,90%
Use of temporary abutments	43	4783	0,90%
Surface treatment	11	4783	0,23%
Region of placement on maxilla	9	4783	0,19%
Other disease	7	4783	0,15%
Use of healing abutment	6	4783	0,13%
Type of graft procedure	5	4783	0,10%
Replacement implant	4	4783	0,08%
Implant loss	4	4783	0,08%
Implant loss flag	4	4783	0,08%
Region_of_placement_on_mandible	3	4783	0,06%
Time_between_bone_graft_and_implant_placement	2	4783	0,04%
Cover_screw	2	4783	0,04%
Adverse_event	2	4783	0,04%
Bone graft procedure	1	4783	0,02%
Use of neodent graft screw	1	4783	0,02%
Tissue graft procedure	1	4783	0,02%
Time between tissue graft and implant placement	1	4783	0,02%
Date of last follow-up	1	4783	0,02%
ID implante	0	4783	0,00%
ID paciente	0	4783	0,00%
Age	0	4783	0,00%
Gender	0	4783	0,00%
Procedure date	0	4783	0,00%
Total implants placed upper and lower arch	0	4783	0,00%
Total Helix implants placed upper and lower arch	0	4783	0,00%

Guided surgery	0	4783	0,00%
Prosthetic interface	0	4783	0,00%
Implant lenght (mm)	0	4783	0,00%
Implant diameter (mm)	0	4783	0,00%

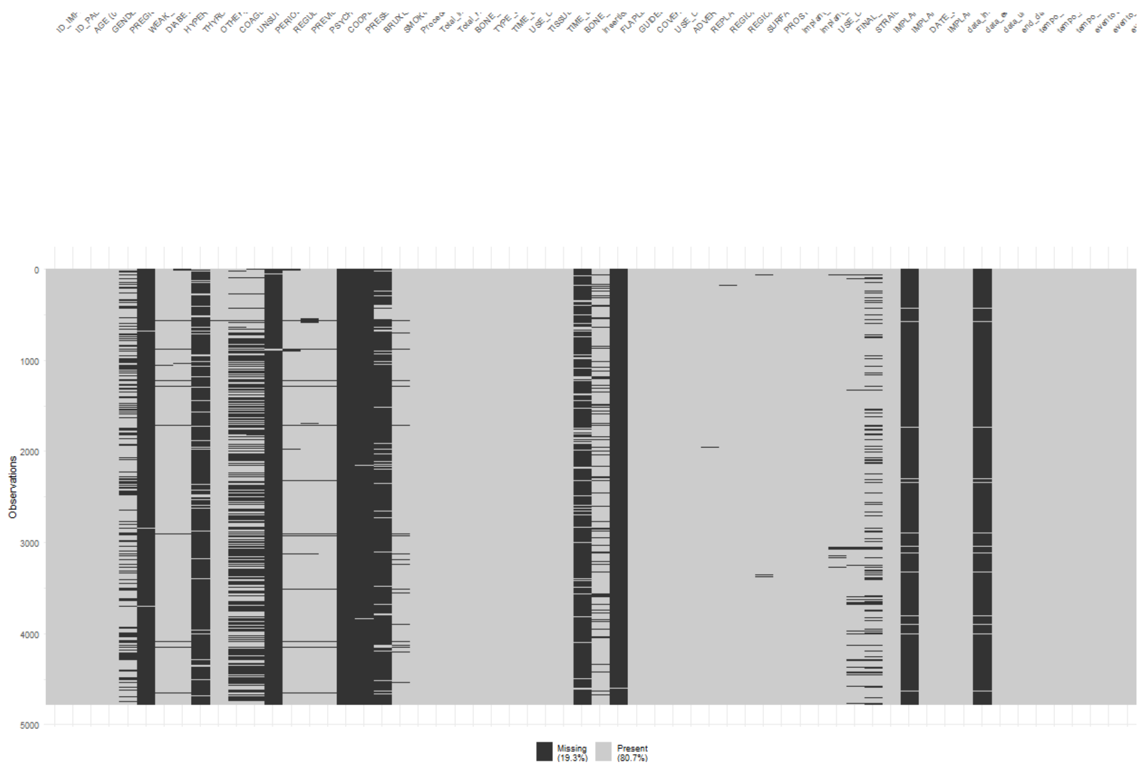

Figure S1 – Missing data map

Table S2 – Descriptive analysis of the patient's characteristics at the patient level (n=1215)

Variable		N	%
Gender	Female	740	60.91
	Male	475	39.09
Pregnancy	No	355	29.22
	Not informed	385	31.69
	Not applicable	475	39.09
Presence of weak immunological system?	Yes	5	0.41
	Not informed	1210	99.59
Diabetes	Yes, controlled diabetes	67	5.51
	Yes, uncontrolled diabetes	2	0.16
	Yes, not informed if controlled	30	2.47
	No	1092	89.88
	Not informed	24	1.98
Hypertension	Yes, controlled hypertension	286	23.54

	Yes, uncontrolled hypertension	7	0.58
	Yes, not informed if controlled	53	4.36
	No	840	69.13
	Not informed	29	2.39
Thyroid disfunction	Yes, controlled thyroid dysfunction	90	7.41
	Yes, not informed if controlled	11	0.91
	No	5	0.41
	Not informed	1109	91.27
Coagulation disorders (hemophilia, low platelet count)	Yes	23	1.89
	Yes, low platelet count	1	0.08
	No, but had bleeding problems in the past	2	0.16
	No	548	45.10
	Not informed	641	52.77
Unsuitable soft tissue capacity?	Yes	16	1.32
	No	553	45.51
	Not informed	646	53.17
Periodontitis	Yes, and treated	13	1.07
	Not informed	1202	98.93
Incomplete jawbone growth	Not informed	1215	100.00
Use of steroids (corticoids)	Yes	73	6.01
	No	1094	90.04
	Not informed	48	3.95
Previously head/neck radiotherapy	Yes, more than 5 years	1	0.08
	Yes, date no informed	2	0.16
	No	1162	95.64
	Not informed	50	4.12
Therapy with bisphosphonate	Not informed	1215	100.00
Presence of xerostomia	Not informed	1215	100.00
Psychological limitations?	Yes	78	6.42
	No	1104	90.86
	Not informed	33	2.72
Cooperative and motivated patient?	No	2	0.16
	Not informed	1213	99.84
Presence of poor oral hygiene?	Yes and treated	2	0.16
	Yes	5	0.41
	No	5	0.41
	Not informed	1203	99.02
Presence of bone metabolism disorders	Not informed	1215	100.00

Bruxism and clenching	Yes	66	5.43
	Yes, use occlusal splint	4	0.33
	Yes, but do not use occlusal splint	1	0.08
	No	37	3.05
	Not informed	1107	91.11
Presence of other diseases?	Yes	506	41.65
	No	706	58.10
	Not informed	3	0.25
Smoking	Yes	56	4.61
	Yes, less than 10 cigarettes/day	50	4.12
	Yes, more than 10 cigarettes/day	36	2.96
	Former smoker	2	0.16
	No	1019	83.87
	Not informed	52	4.28

Table S3. Descriptive analysis of surgical procedure variables at implant level (n=4783).

**Variable**		**N**	**%**
Bone graft procedure	Yes	1295	27.08
	No	3487	72.90
	Not informed	1	0.02
Type of graft procedure	Autogenous	32	0.67
	Synthetic	1	0.02
	Xenogenous	1256	26.26
	Not informed	6	0.13
	Not applicable	3488	72.92
Time between bone graft and implant placement	In conjunction with implant placement	927	19.38
	1-4 months	15	0.31
	5-6 months	10	0.21
	7-12 months	156	3.26
	More than 1 year	186	3.89
	Not informed	2	0.04
	Not applicable	3487	72.91
Tissue graft procedure	Yes	809	16.91
	No	3973	83.07
	Not informed	1	0.02
Time between tissue graft and implant placement	In conjunction with implant placement	430	8.99
	1-4 months	31	0.65
	5-6 months	19	0.40
	7-12 months	155	3.24
	More than 1 year	174	3.64

	Not informed	1	0.02
	Not applicable	3973	83.06
Bone type	I	46	0.96
	II	215	4.50
	III	232	4.85
	IV	44	0.92
	Not informed	4246	88.77
Implant loading type	Immediate	2302	48.13
	Conventional	1735	36.27
	Not informed	746	15.60
Insertion torque (N.cm)	<= 10	80	1.67
	>10 and <32	572	11.96
	32-60	3213	67.18
	>60	245	5.12
	No torque	1	0.02
	Not informed	672	14.05
Flapless of open flap surgery	Flapless	9	0.19
	Open flap	44	0.92
	Not informed	4730	98.89
Guided surgery	Yes	474	9.91
	No	4309	90.09
Region of implant placement on maxilla	Incisor	530	11.08
	Canine	174	3.64
	Premolar	823	17.21
	Molar	702	14.68
	Full arch	218	4.56
	Not informed	9	0.19
	Not applicable	2327	48.64
Region of implant placement on mandible	Incisor	113	2.36
	Canine	29	0.61
	Premolar	461	9.64
	Molar	1120	23.42
	Symphysis	1	0.02
	Full arch	607	12.69
	Not informed	3	0.06
	Not applicable	2449	51.20
